# Exploring usability metrics in continuous glucose monitoring systems: insights from the voice of people with diabetes in Italy

**DOI:** 10.3389/fcdhc.2025.1472471

**Published:** 2025-03-13

**Authors:** Martina Manzoni, Davide Minotti, Giovanni Toletti, Andrea Boaretto

**Affiliations:** ^1^ Department of Management, Economics and Industrial Engineering, Politecnico di Milano, Milan, Italy; ^2^ Personalive srl, Milan, Italy

**Keywords:** continuous glucose monitoring, usability indicators, diabetes management, patient perspectives, user satisfaction, quality of life, healthcare technology, patient-centered care

## Abstract

**Introduction:**

Continuous Glucose Monitoring (CGM) systems are crucial in diabetes management, offering clinical and psychological benefits despite operational challenges. Usability assessment of real-time and intermittently-scanned CGM systems is a notable research gap. This study, in collaboration with diabetes patient associations, explores CGM usability from the perspective of Italian individuals with diabetes.

**Methods:**

A roundtable discussion with patient association representatives was conducted to discuss CGM usability, followed by a detailed online survey of 281 Italian patients on CGM usage, satisfaction, and feature preferences.

**Results:**

Findings show a significant positive impact on Quality of Life (87/100) and moderate usability (66/100). Core CGM functions are widely used, while data sharing with healthcare professionals is underutilized. The study offers diverse insights into CGM usability from both the roundtable and survey data.

**Conclusions:**

The study underscores the importance of CGM in diabetes management and highlights the need for continuous technological improvements. It emphasizes the role of patient associations in enhancing communication with manufacturers and CGM education. Effective collaboration between healthcare professionals and patients is vital for optimal CGM use, advocating for personalized care strategies tailored to individual patient needs.

## 1 Introduction

Diabetes mellitus, a chronic metabolic disorder characterized by frequent blood glucose fluctuations, is a global health crisis. The International Diabetes Federation (IDF) estimates that 537 million people had diabetes in 2021, projected to reach 783 million by 2045 (1). The implications of this disease are profound, with diabetes contributing significantly to the population health and healthcare costs (2). Managing diabetes involves maintaining near-normal glucose levels to prevent complications (3). Traditional self-monitoring via fingerstick tests has limitations, such as discomfort and lack of continuous data, prompting the search for innovative solutions.

Technological advancements, like insulin pens, pumps, and portable glucometers, have transformed diabetes management. Continuous Glucose Monitoring (CGM) systems represent a significant breakthrough. CGM systems include intermittently scanned CGM (is-CGM) and real-time CGM (rt-CGM). Is-CGM requires manual scanning to view glucose data, while rt-CGM offers continuous real-time data (4). This distinction allows rt-CGM users to respond swiftly to glycemic fluctuations, while is-CGM suits those preferring less intrusive monitoring.

The clinical and psychological benefits of CGM systems are well-documented (5–7), especially for insulin-treated patients (8, 9). Understanding the features that promote sustained CGM use is crucial. While one study explored CGM usability satisfaction (10), evidence on specific features like self-reliance sensors (11) and real-time glucose values (12) remains sparse. Addressing user needs through multiple features is vital for optimizing CGM technology, enhancing user satisfaction, and adherence for better therapeutic outcomes. Few studies analyze patient perspectives on CGM usability and satisfaction, although patient input is essential for advancements (10).

This study examines the perspectives of Italian individuals on CGM systems, aiming to identify usage patterns, key usability parameters, satisfaction levels, and Quality of Life impacts. It also investigates potential perception disparities across patient cohorts based on age, diagnosis duration, sensor use duration, and sensor type (rt-CGM or is-CGM).

## 2 Materials and methods

**Figure 1:**
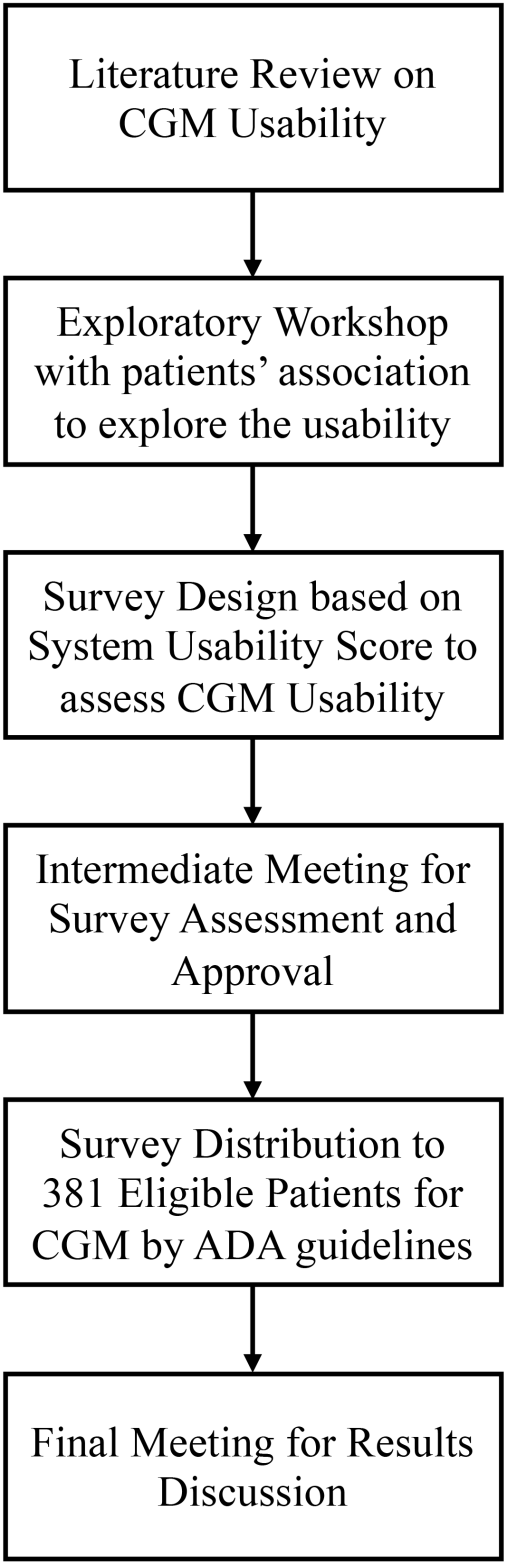
Research steps.

The methodology for this study followed a multi-step approach, encompassing several key phases to gather comprehensive data and insights on the usability of CGM systems from Italian individuals with diabetes. The steps included a preliminary narrative literature review of existing literature on CGM devices. Afterwards, the following steps took place, as shown in **Figure 1**.

1. Exploratory Workshop: An initial workshop with representatives from diabetes patient associations to discuss CGM usability.
2. Survey Design: Development of the survey questions through a co-creation process, integrating input from the workshop.
3. Intermediate Meeting: A follow-up meeting with patient association members to refine the survey based on their feedback.
4. Survey Distribution: Administration of the survey to eligible patients and collection of their responses.
5. Final Meeting: Presentation of survey results to patient associations and discussion of future research opportunities.

### 2.1 Data collection and analysis

The aforementioned workshop was held in a roundtable format and involved representatives from major Italian patient associations. During the event, they were asked to present their definitions of usability for a CGM system. Following this, they matched different patient profiles (in the form of personas) with the CGM functionalities that best met their needs. This workshop functioned as an exploratory analysis, providing insights that informed the thematic structure of the survey.

Subsequently, an *ad hoc* survey was developed in detail. Such survey includes both questions arising from the reflections discussed during the workshop, in a co-creation logic and questions retrieved from extant studies, specifically form the System Usability Scale (SUS) (13). It was meticulously structured to collect comprehensive data on participants’ diabetes histories, current CGM usage, and their experiences with these devices. A multifaceted approach was adopted, incorporating multiple-choice questions, Likert-scale queries, and open-ended interrogatives, facilitating the accumulation of both quantitative and qualitative data.

System Usability Scale (SUS) (13) was employed to assess the overall usability dimension connected to the CGM device’s associated application. This tool, rooted in usability assessment and user experience research, comprises ten questions that provide insights on various usability facets, such as ease of use, learnability, and user satisfaction. Respondents rate each question on a Likert scale from 1 to 5, with 1 indicating strong disagreement and 5 indicating strong agreement. The scores are aggregated to produce a SUS score ranging from 0 to 100, indicating the overall usability of the digital product. A high SUS score signifies enhanced usability, whereas a low score indicates usability deficiencies requiring remediation (14). It is important to note that the usability characteristics assessed in this study were general and not tied to any specific CGM device. While each CGM device may have unique features, usability aspects are largely transversal. Our approach aimed to abstract the impact of these characteristics on usability to derive general guidelines, rather than focusing on the evaluation of individual devices. The survey transcript is outlined in Supplement 1.

In the third step, a meeting with the same patient associations’ representatives was held, where the rationale behind the design of the survey was explained. Taking inspiration from the feedback provided by the associations’ members, the survey was enriched and expanded to include their suggestions and insights.

Following this, the survey was administered to eligible patients. Participants were recruited through collaborative initiatives with Italian patient associations, which used their channels to distribute the questionnaire among diabetic individuals, who could complete it voluntarily. Patient associations played a crucial role throughout the study, ensuring the representativeness of the sample by identifying suitable respondents and spreading the survey uniformly across the Italian territory and different age groups.

Data acquisition was executed anonymously through an online survey platform, employing a Computer-Assisted Web Interviewing (CAWI) modality. Participants were apprised of the survey’s objectives and procured informed consent prior to their participation. Eligibility criteria necessitated a prior diagnosis of diabetes, insulin dependency in diabetes treatment and ongoing utilization of a CGM system. Caregivers of eligible respondents were allowed to record their answers as well, responding on behalf of patients, who mostly represented their offspring.

Regarding data analysis, quantitative data underwent scrutiny employing SPSS statistical software, with descriptive statistics such as frequencies and percentages thoughtfully harnessed to capture response trends. Qualitative data arising from open-ended inquiries were subjected to thematic analysis, discerning recurring topics and patterns within the narratives. In pursuit of a comprehensive examination of nuanced preferences within subgroups, cross-analyses were conducted, utilizing two key metrics: the percentage difference between each subgroup cluster and the baseline and the Affinity Index (AI) measuring the degree of association of the observed phenomena with the reference group. The subgroups selected to gather nuanced preferences were age, diagnosis duration, sensor use duration and sensor type.

In the final stage, a concluding meeting with the patient associations involved in the study was held to present the questionnaire’s results. Joint considerations over the findings emerged, discussing their implications for future research.

## 3 Results

### 3.1 Narrative review of the literature

This section provides an overview of existing literature on CGM devices, highlighting their ambivalent effects and the challenges patients face.

As outlined by Kang et al. (2022), *“CGM reframes diabetes self-management”* (8). These systems encompass a distinctive array of attributes that enhance the capacity for proactive and efficacious diabetes management, surpassing the capabilities inherent in capillary blood glucose monitoring (BGM) modalities (15).

Particularly noteworthy among the constellation of attributes contributing to user satisfaction within the realm of CGM systems is the permanent accessibility to real-time glucose values. This convenience obviates the necessity for the discomfort-inducing ritual of finger-pricking, a procedure obligatory in the case of traditional BGM systems (16). Moreover, this feature also serves to mitigate undue attention to their actions, a crucial concern, especially in public domains such as workplaces, during travel, at public events, or while operating vehicles.

Furthermore, the inclusion of trend arrows in CGM systems assumes paramount significance, as it endows patients with the ability to intuitively discern anticipated fluctuations in glucose levels, along with predictive trajectories of such changes. This empowers patients to mount timely responses to diurnal glycemic oscillations, including those of unforeseen provenance, thereby preventing the threat of severe hyperglycemic and hypoglycemic episodes (12).

Finally, the cornucopia of data made accessible through the utilization of CGM augments patients’ comprehension of the intricate interplay between their lifestyle choices and pharmacological interventions regarding blood glucose levels (8, 11). This perspicacity equips individuals to discern the ramifications of daily activities such as dietary regimens, physical exertion, and medication adherence on glycemic trends. Consequently, it not only enables therapeutic adjustments but also fosters a heightened awareness, motivating proactive decisions aimed at refining metabolic control.

Despite their transformative potential, CGM systems are not devoid of challenges. People with diabetes report concerns regarding accuracy and ambivalent opinions about certain device functionalities, which can undermine trust and, consequently, adherence to these technologies (12).

Accuracy discrepancies between CGM readings and traditional fingerstick measurements have been a focal point of scholarly inquiry. This issue remains a critical concern (17, 18), necessitating continuous technological refinement. Additionally, the overwhelming influx of real-time data poses challenges related to information overload and data interpretation (19). Alarms can be a source of annoyance and frustration, known as alarm fatigue (20). Simultaneously, the use of wearable technology like CGM tends to draw attention to the presence of the sensor, generating anxieties and concerns about image and attractiveness (21).

In conclusion, the available literature on this topic highlights the achievements in autonomous diabetes management thanks to CGM systems but also recognizes numerous open challenges. While patients greatly benefit from CGM technology, there are still limitations related to concerns and unmet needs that can negatively impact the attainment of expected therapeutic goals. Most barriers to treatment continuity, such as alarm fatigue or information overload, are related to the concept of usability, which refers to the extent to which a product can be used by users to achieve specific objectives with effectiveness, efficiency, and satisfaction (22). When applied to a medical technology like a CGM system, the concept of usability becomes an integral part of the individual and therapeutic journey, in collaboration with healthcare professionals. Human factors and user-centric design principles have garnered significant attention in recent research endeavors, emphasizing the influence of device ergonomics, data visualization interfaces, and user experience on long-term adherence and glycemic control. In light of the significance of glucose monitoring instruments in the enhancement of individuals’ Quality of Life, the incorporation of usability considerations is crucial to optimize the consistent utilization of these devices, thereby endeavoring to attain the utmost clinical and psychological advantages.

### 3.2 Survey results

Following the literature review, an initial workshop with representatives from diabetes patient associations was conducted to discuss CGM usability. During this workshop, the major topics about usability that emerged from the literature were discussed in detail. Subsequently, the survey was tested and validated to ensure that all questions were clear and comprehensible to the participants.

A total of 381 responses were collected during the survey, with 41 responses marked as incomplete. Subsequently, 56 participants using traditional glucometers were excluded from the analysis. Ultimately, 284 participants who used CGM systems were considered eligible for the study. After omitting three insulin pump users who do not interpret glucose values or self-administer insulin, the final sample consisted of 281 participants. **Table 1** details the sample distribution by demographics (age, gender, geographic origin) and clinical parameters (diabetes type, treatment methods, and diagnosis duration).

**Table 1:** Socio-demographic data of the sample (N=281).

Variable	Responses (#) N = 281	Responses (%)
Gender		
Male	133	47%
Female	147	52%
I prefer not to say	1	1%
Age range		
<18	67	24%
18-44	79	28%
45-64	101	36%
65 and over	34	12%
Geographical Area		
Northeast	92	33%
Northwest	72	26%
Center	54	19%
South	63	22%
Diabetes type		
Type 1	265	94%
Type 2	13	5%
Other	3	1%
Treatment		
Insulin	275	98%
Basal insulin	4	1,3%
Non-insulin medications	2	0,7%
Diagnosis duration		
< 6 months	25	9%
6 months – 1 year	28	10%
1 – 2 years	52	18%
> 2 years	176	63%
Type of sensor		
rt-CGM	196	70%
is-CGM	85	30%

The sample exhibited a balanced gender distribution and comprehensive geographic representation across Italy. Most participants had Type 1 diabetes and managed it with multiple daily insulin injections. Although this sample design might appear to reduce the statistical power, it was deliberately chosen to align with ADA guidelines (4), which recommend that real-time continuous glucose monitoring or intermittently scanned continuous glucose monitoring be offered to adults with diabetes who are on multiple daily injections or continuous subcutaneous insulin infusion and are capable of using these devices safely. Consequently, we focused on collecting usability information from this specific group to ensure the findings were directly applicable to the target population. This group also represents those eligible for CGM reimbursement within the Italian healthcare framework, where the survey was conducted. Notably, nearly 75% of the participants had been living with diabetes for over two years.

### 3.3 Usability of the sensor

The ensuing section embeds an exploration of the usability dimensions associated with CGM sensors, as employed by the surveyed cohort. This analysis encompasses a comprehensive assessment of the physical characteristics of these sensors, the simplicity and efficacy of the procedures they entail, and the nuanced psychological ramifications stemming from the act of wearing those sensors.

Before going in depth on such analysis, it is noteworthy that to these participants relying on traditional glucometers, some additional questions were asked. However, as the sample size was limited, it was not possible to compare results or have statistically significant insights. However, it is relevant to note that approximately 89% of respondents who use BGM indicated awareness of the possibility of measuring glucose using Continuous Glucose Monitoring (CGM) devices or sensors. Additionally, among the 47 respondents who showed awareness of CGM devices, 21 individuals (45% of the aware sample) stated that they would definitely or very likely adopt a CGM device if recommended by their physician.

#### 3.3.1 Sensor attributes and procedures

The empirical examination of usability indicators has yielded noteworthy findings, underscoring the sample’s discerning focus on specific sensor attributes, as delineated in descending order of significance in **Table 2**.

**Table 2:** Feature importance and benchmark with subgroups.

Feature Importance	is-CGM					rt-CGM				
	1	2	3	4	5	1	2	3	4	5
Waterproofing	1,5%	1,5%	22,1%	38,2%	36,8%	0,0%	2,1%	10,3%	29,5%	58,2%
# of authorized positions	2,9%	20,6%	32,4%	26,5%	17,6%	1,3%	14,1%	21,5%	40,9%	22,1%
Thickness	4,4%	20,6%	27,9%	30,9%	16,2%	4,1%	13,5%	34,5%	29,7%	18,2%
Dimension	2,9%	20,6%	30,9%	26,5%	19,1%	2,7%	19,3%	36,7%	24,7%	16,7%
Weight	10,3%	26,5%	20,6%	22,1%	20,6%	10,0%	22,7%	22,0%	29,3%	16,0%
Aesthetics	20,6%	36,8%	22,1%	11,8%	8,8%	22,0%	42,0%	20,7%	9,3%	6,0%

Scale used: 1. Not at all important; 2. Slightly important; 3. Fairly important; 4. Very important; 5. Extremely important.

It is readily discernible from this hierarchical prioritization that these enumerated functionalities are instrumental in satisfying two distinct patient requisites. First, they cater to the demand for a wearable device that obviates issues of physical discomfort or dermal adhesion, thereby conferring a seamless and nonintrusive experience. Second, they underscore the pivotal significance accorded to the number of permissible positions for sensor placement, thus endowing patients with the opportunity to select the most appropriate anatomical place, considering both daily activities (e.g., occupations that expose the sensor to water, dust or mechanical impacts) and discretion requisites.

These imperatives blend within the concept of device portability, encompassing dual facets: wearability and cutaneous comfort on one hand, and sensor ergonomic considerations, contributing to discretion, on the other.

When delving into subgroup analyses, it becomes evident that users of rt-CGM devices assign heightened importance to multifaceted sensor placement (+19 percentage points compared to intermittent scanning CGM users) and waterproofing (+13 percentage points compared to is-CGM users, as highlighted in **Table 2**.

In the evaluation of the procedural ease associated with sensor application and activation, an array of parameters was scrutinized. The process of sensor attachment to the body is perceived as easy or extremely easy by two-thirds of the sample.

The interconnectivity with smartphones is categorized as easy or extremely easy in 51% of instances. Predominant technical difficulties encountered encompass defective sensors (42%), suboptimal durability (30%), dermal irritations (28%), compatibility issues (28%), and high alarm frequencies (14%) – (i.e., the percentages reported in the results section are calculated based on the overall number of respondents, indicating the proportion of respondents who selected each specific option, regardless of whether they chose multiple items). It is noteworthy that in our study the dermal irritation are reported in a lower frequency than in extant literature (23). However, it is important to note that recent research has identified IBOA as a main allergen in such cases (24). With the recognition of IBOA as a potential allergen, companies have initiated efforts to produce patches without IBOA or develop solutions to mitigate skin issues associated with CGM device use. As our data are relatively recent, it is possible that advancements in technology and formulation have led to a lower prevalence of skin issues in our study population. These findings corroborate the compelling requisites of physical comfort and sensor robustness that were previously evidenced in the literature.

#### 3.3.2 Emotional responses to CGM sensors

Further digging into the more introspective dimensions of the CGM system-user relationship, 67% of respondents articulate feelings of safety, 44% as recipients of care, and 56% as beneficiaries of protection, with scores spanning a range from 3.6 to 3.8 on a 5-point scale. Conversely, three negatively framed assertions—”I feel judged,” “I feel anxious,” and “I feel troubled”—attain medium to modestly low ratings (all less than or equal to 2 on a 5-point scale), with over three-quarters of respondents indicating minimal or negligible levels of perceived judgment, anxiety, or distress. These findings suggest that, on the whole, currently employed CGM devices are efficacious in delivering the requisite levels of reliability necessary to inculcate trust in the technology.

Individuals recently diagnosed with diabetes exhibit an 8% higher likelihood of feeling judged in relation to sensor usage, a phenomenon partially attributable to incomplete acceptance and comprehension of diabetes, a facet more robustly developed in patients with longer-standing diagnoses.

Caregivers of individuals under 24 years of age, instead, are worth a separated discourse. Indeed, they tend to register lower perceptions in terms of feeling cared for (-8 percentage points), secure (-14 percentage points), and protected (-12 percentage points), thus underscoring the discernible impact that influential figures such as parents, have in shaping the daily management of diabetes (1). Parents often exhibit a distinct perception of diabetes and diabetes-related technology in contrast to that of their offspring. Their concerns typically center around the well-being of their children, and this inherent worry can provoke a sense of apprehension and skepticism towards CGM systems employed by the patients. **Table 3** shows the detailed comparison between the baseline and the abovementioned cluster.

**Table 3:** Emotional sentiments and benchmark with caregivers of children and adolescents.

Emotional sentiment	Baseline: average	Baseline: percentage of 4 and 5	Caregivers of <24 patients: percentage of 4 and 5
Secure	4,3/5	67%	52.6%
Protected	3,7/5	56%	43.9%
Cared for	3,4/5	53.7%	45.6%

### 3.4 Usability of the mobile application

The ensuing section embeds an exploration of the usability dimensions associated with CGM sensors, as employed by the surveyed cohort. This analysis encompasses a comprehensive assessment of the physical characteristics of these sensors, the simplicity and efficacy of the procedures they entail, and the nuanced psychological ramifications stemming from the act of wearing those sensors.

#### 3.4.1 Usage frequency of the application and its functionalities

In regard to usage frequency, the application emerges as a pivotal instrument in the realm of daily diabetes management, with a commanding 63% of respondents accessing it five or more times daily for glucose level monitoring, with no particular difference between the is-CGM and rt-CGM users, as shown in **Table 4**.

**Table 4:** App Access frequency.

App Access frequency	is-CGM	rt-CGM
2-3 times per week	2,9%	5,3%
1-2 times per day	2,9%	6,0%
3-4 times per day	13,2%	13,3%
5 or more times per day	64,7%	62,7%
Other	16,2%	12,7%

Delving into the usage frequency of specific app functions, it becomes apparent that, notwithstanding the incorporation of a multitude of features within respondents’ applications, certain functionalities are employed more frequently. These functionalities encompass the visibility of glucose levels (utilized by 85% of patients on a daily or near-daily basis), the availability of trend indicators, and the configuration of diverse alarm types—ranging from threshold-triggered alarms to predictive alerts and signal loss warnings.

Core functionalities, therefore, emerge as the most leveraged by users. Paradoxically, the capacity to share monitoring data with a healthcare professional is seldom or never utilized by 40% of respondents. **Table 5** displays those with a high usage frequency prevalence (30% or more).

**Table 5:** Presence and usage frequency of different functions within CGM applications.

Function	Function presence percentage	High usage frequency (every day or almost every day) percentage
Trend arrows	99%	85%
Visibility of glycemic value	95%	85%
Predictive alarms	87%	37%
Lost signal alert	89%	35%
Alarms	90%	32%
Visibility on other parameters (TIR, history of glycemic values)	98%	31%
Possibility to note events (e.g., carbohydrate intake, physical activity)	87%	30%

Subsequent sub-analyses unveil that users of rt-CGM devices tend to employ alarm settings, predictive alarms, signal loss alarms, alarm delay mechanisms, and follower mode to a greater extent than is-CGM users, thereby aligning with their choice of a more technologically advanced apparatus.

Moreover, the application’s usability was subjected to analysis through the application of the System Usability Score (SUS). The CGM monitoring system applications employed by respondents garnered an aggregate SUS score of 66 out of 100, indicative of a satisfactory perception of usability, albeit lacking any conspicuous waves of excitement.

#### 3.4.2 Co-creation of the ideal CGM application

The objective of the ensuing paragraph is to undertake a comparative analysis between findings arising from roundtable discussion with patient associations and survey responses from diabetes patients, aiming to delineate diverse perspectives on essential functionalities for the ideal CGM application.

During ideation phase, concerning the concept of usability, intriguing perspectives emerged, shedding light on the diverse interpretations held by patient associations with respect to the usability of a CGM application.

Notably, simplicity and intuitiveness were emphasized, advocating clear indicators for glucose values, trends, and active insulin dosages. Conversely, some representatives argued for a comprehensive array of functions, perceived as critical in guiding patients throughout their therapeutic journey and daily life, spanning physical activity management, dietary recommendations, bolus corrections, carbohydrate counting, and seamless integration with insulin delivery devices. Universal compatibility with mobile phones and accessibility across various devices, such as personal computers and tablets, were underscored as transversal usability elements.

Moreover, during the roundtable discussion, a primary emphasis was the identification of pivotal functionalities tailored to specific patient profiles, postulating three personas (see **Table 6**). For individuals averse to their diabetes diagnosis and fingerstick tests, predictive alarms, trend arrows, and lost signal alerts were paramount. Engaged athletes, characterized by a propensity to forget periodic glucose measurements and an aversion to disrupting their scholastic or social commitments, prioritized non-invasive alarms, waterproofing, and integration with insulin pens. Anxious individuals, intensely dedicated to diabetes management, emphasized functionalities like first alert delay, adjustable hyperglycemic thresholds, especially in the postprandial context, and suitable recommendations for insulin dose adjustments. These nuances underscore the imperative for highly customizable CGM applications to cater comprehensively to the diverse and intricate end-user demands.

**Table 6:** CGM functions selected by patient associations’ representatives as the most suitable for three different patient profiles.

Profile	Most suitable CGM functions
Female, 65 years old, Type 2 diabetes for 7 years, Multiple Daily Insulin Injections (MDI) and SMBG, averse to diabetes diagnosis and fingerstick tests	Predictive alarms Trend arrows Lost signal alert
Male, 20 years old, Type 1 diabetes for 6 years, MDI and intermittently-scanned CGM, perpetually engaged athlete	Non-invasive and discrete alarms Predictive alarms Waterproofing Integration of the CGM system with the insulin pen
Female, 41 years old, Type 1 diabetes for 12 years, MDI and real-time CGM, anxious and intensely dedicated to the management of diabetes,	First alert delay Lost signal alert Adjustable hyperglycemic thresholds Recommendations for insulin dose administration

In the survey, instead, patients were asked to indicate the three core functions they would include in the ideal CGM Mobile application. Results reflect a consensus on essential features intrinsic to all CGM systems as the real-time display of glucose values and trends for daily monitoring, occupy the zenith of their enumeration, endorsed by 61% and 50% of patients, respectively. Smartphone compatibility, specifically 30% for Android and 20% for iOS, and data sharing capabilities with healthcare professionals (22%) were subsequent priorities. Other attributes, including additional features related to therapy management, received varying levels of enthusiasm, with endorsements equal to or less than 13%. Furthermore, the survey highlighted a widespread inclination for additional features within the CGM application addressing therapy management needs. These features encompassed information on active insulin administration and insights into lifestyle aspects, encompassing dietary choices and physical activity regimens.

This comparative analysis elucidates multifaceted perspectives on the ideal CGM application. While simplicity and intuitive design remain fundamental, the diversity of patient needs necessitates a highly customizable approach.

### 3.5 Satisfaction and impact on quality of life

This section aims at unveiling the satisfaction and perceived impact on Quality of Life of the CGM systems in use by the sample.

Patient satisfaction with CGM systems is medium-high (3,9 out of 5 as an average), with 68% of the respondents reporting a high or extremely high level of satisfaction. Satisfaction with the application, instead, is slightly lower (3,4 out of 5). **Table 7** reports satisfaction items and percentages for the sensor and the application. It has been ascertained that individuals assuming the role of caregivers for pediatric and adolescent patients tend to exhibit a comparatively lower level of satisfaction (-4,2 percentage points). This observed diminishment is in consonance with the concomitant diminution in the level of trust experienced by individuals who undertake the daily responsibility of patient care, yet do not themselves utilize CGM sensors.

**Table 7:** Patient satisfaction benchmark between CGM sensor and application.

Item	Satisfaction with the sensor	Satisfaction with the application
Not satisfied at all	0%	1%
Not very satisfied	3%	8%
Somewhat satisfied	29%	46%
Very satisfied	49%	35%
Extremely satisfied	19%	10%

Notably, a substantial 87% of the survey participants attested to the favorable impact of the extant CGM system on their QoL. Upon conducting nuanced sub analyses, this favorable perception is observed to exhibit a more pronounced relationship with users who categorize the sensor as highly or extremely secure (+22 percentage points, AI 140), those who report a very high or extremely high level of satisfaction (+27 percentage points, AI 141), and those who manifest an augmented sense of being cared for (+17 percentage points, AI 132), secure (+19 percentage points, AI 128), and protected (+22 percentage points, AI 142). These empirical findings underscore the pivotal role played by individual feelings and perceptions in shaping a positive evaluation of the CGM device’s impact on Quality of Life.

## 4 Discussion

Diabetes mellitus represents a significant global health challenge with profound implications for individuals and healthcare systems alike (1). CGM systems have emerged as transformative tools in the daily management of diabetes, significantly improving health outcomes (25–27) and proving to be a cost effective solution with respect for SMBG (28) when consistently used. To foster the utilization of CGM and then to optimize clinical outcomes, our study aims at shedding novel light on what characteristics better perform among diabetic patients.

Usability-related elements of CGM systems emerged as a central theme in this study, in line with extant research (29). The hierarchy of attributes prioritized by respondents reflects the intricate interplay between wearability, comfort, and sensor performance to overcome usability barriers (30). Waterproofing and the ability to place the sensor in multiple positions stand out as paramount features of the sensor. These functionalities not only enhance user comfort but also offer practical advantages, such as the ability to wear the sensor during various activities without discomfort or fear of detachment. It is notable that adolescents, who are often more conscious of appearance, show a heightened preference for these attributes. Moreover, the dimensions, proportions, and mass of the CGM sensor also influence user satisfaction, particularly among those recently diagnosed or with shorter sensor usage durations. This highlights the evolving preferences of individuals as they gain experience with the technology.

The procedural ease of sensor application and activation was generally perceived as straightforward by most respondents. Conversely, patients recognize minor concerns linked to sensor adhesiveness and resistance. These findings echo the worries raised in previous research and underscore the need for continued improvement in sensor robustness (8).

Concerning the emotional sentiments associated with CGM system usage, most respondents expressed feelings of security, care, and protection while using CGM devices. These sentiments reflect the role of CGM technology in instilling trust and confidence in individuals with diabetes. Notably, feelings of judgment, anxiety, or distress were rare, indicating that CGM systems generally succeed in creating a supportive and non-judgmental environment for users.

The usability of mobile applications linked to CGM systems is a critical aspect of the overall user experience. The present findings reveal that the application is frequently accessed, underscoring its essential role in daily diabetes management. Users exhibit varying patterns of app feature utilization; with core functionalities such as real-time monitoring and trend indicators being most commonly used.

This study provides an exposition of viewpoints pertaining to the subject matter, drawing from both patient associations and individuals utilizing CGM technology. The analysis serves to underscore the conspicuous disparities in the conceptualization of usability within this context (11). Representatives from patient associations, characterized by a heightened level of diabetes-related knowledge in comparison to the average diabetic population, offer divergent viewpoints regarding the quantity and the specific functionalities that engender usability in a CGM application. Conversely, patients themselves articulate a desire for and recognition of the value associated with supplementary features concerning therapeutic management within a CGM application. Nevertheless, they concede to a predominant reliance on the core functionalities, with a tendency to disregard the array of supplementary features already at their disposal.

One of the most notable findings of the study is the overwhelmingly favorable impact of CGM systems on individuals’ Quality of Life, in line with the positive outcomes showed in past research (31). A remarkable 87% of participants reported a positive influence, and this perception was even more pronounced among users who felt secure, satisfied, cared for, and protected. These results underscore the crucial role of CGM technology in enhancing overall well-being, empowering individuals with diabetes to lead more confident and fulfilled lives.

The findings presented in this research have significant implications for CGM producers, patient associations, and healthcare professionals in the context of diabetes management. More specifically, this study highlights the need to solve patients’ needs with the proper glucometer features, in order to foster the continuous utilization of the device and ensure the long-term benefits that have been widely shown in extant literature (32, 33). It is therefore paramount to understand which characteristics are the drivers upon which the CGM should be advised by healthcare professionals to the patients.

For CGM producers, the emphasis on wearability, comfort, and sensor performance underscores the importance of continually improving the design and functionality of their devices (34). Waterproofing and versatile sensor placement are key features that enhance user comfort and practicality, particularly among adolescents who prioritize appearance. Manufacturers should consider the multitude of patient profiles and the evolving user preferences, with the aim to tailor the CGM technology on each individual. This personalization may be achieved either by adding valuable features to CGM devices or by removing functions that are not employed or considered redundant.

Patient associations play a pivotal role in championing the cause of individuals afflicted by diabetes. This study acknowledges their profound expertise within the diabetes domain, which drives them to advocate for the advancement of cutting-edge CGM functionalities. However, representatives of patient associations, characterized by a heightened level of diabetes-related knowledge in comparison to the average diabetic population, offer divergent viewpoints with respect to patients. As a matter of fact, the findings from the survey visibly indicate that daily CGM usage predominantly centers on basic and core device features. This discrepancy suggests a need for collaboration between these two groups to ensure that CGM applications meet the multitude of needs and preferences of the diabetic community, and to enable the CGM systems to fit both basic and advanced user requirements. Patient associations, with their in-depth knowledge, can help bridge the gap between individuals and technology and provide valuable input to manufacturers in order to device personalization.

Healthcare professionals already recognize the overwhelmingly positive impact of CGM systems on individuals’ Quality of Life (35). The sense of security and protection offered by CGM devices instills trust and confidence in patients, reducing feelings of anxiety or distress. Nevertheless, it is imperative to recognize the unexpectedly limited utilization of data sharing functionality among patients. The observed underutilization of the functionality may be attributed to the degree of integration of digital channels within the patient care pathways at respective clinical settings. Specifically, the absence of such integration may lead physicians to dissuade patients from transmitting data, anticipating challenges in its incorporation during subsequent visits and the potential for fostering unrealistic patient expectations. This revelation accentuates the imperative for effective communication between patients and healthcare practitioners, emphasizing the utilization of diverse communication channels, while mitigating the individual burden on physicians.

In addition to encouraging CGM technology adoption, healthcare professionals play a crucial role in selecting the CGM system they consider more suitable to the specific needs and requirements of every patient profile. Additionally, they should offer explicit guidance on CGM application usage to cultivate a collaborative environment contributing to the effective attainment of therapeutic goals.

In summary, the research highlights the need for CGM producers to continue innovating their products by selecting the right set of features for every individual, patient associations to advocate for user-centric design and continue supporting patients, and healthcare professionals to effectively promote the adoption of CGM technology. CGM systems have the potential to significantly improve the lives of individuals with diabetes, and working on their usability can help ensure that these benefits reach a broader patient population (15).

This study, while valuable, faces limitations. Predominance of Type 1 diabetes participants, albeit the prerequisite of insulin therapy forces to include more Type 1 patients, may limit broad applicability. Lack of detailed clinical histories, due to privacy concerns, constrains analytical depth and contextualization. Absence of lifestyle data hampers nuanced insights into participant needs. Moreover, perspectives from healthcare professionals were absent, limiting the breadth of the research. These limitations should be meticulously weighed when contemplating the implications and generalizability of the research findings, as they undeniably limit the breadth and transferability of results and conclusions.

Additionally, the focus of the survey was broad, encompassing very different topics about the usability of the device. While this comprehensive approach bolstered the breadth of our data collection, we acknowledge that it precluded an exhaustive exploration of each facet. Nonetheless, themes emerging from the literature review or referenced within the survey warrant deeper investigation. Future research endeavors could explore these areas more comprehensively, focusing on aspects such as the impact of perceived accuracy and performance, as well as the role of alarms in fostering patient trust in the device. While this study highlights the key characteristics necessary to enhance system usability, future investigations could provide a more detailed examination of how these features might be improved, systematically integrated into the device, and optimized to address both the needs and preferences of end users. Such research would contribute to the development of more effective and user-centered solutions in the field of continuous glucose monitoring systems.

## 5 Conclusion

In conclusion, this research endeavor furnishes substantive insights into the metrics of usability and the consequential impacts of Continuous Glucose Monitoring (CGM) systems upon individuals handling diabetes.

In particular, it underscores the existence of latent opportunities for further enhancement, particularly regarding sensor durability and cross-device compatibility. In the inexorable progression of diabetes management, collaborative synergy among patients, healthcare professionals, technology developers, and patient associations emerges as the pivot for optimizing CGM systems to strengthen usability and augment the overall Quality of Life.

These empirical findings possess the potential to inform the trajectory of development and refinement in the realm of CGM systems, with the ultimate objectives of heightening user satisfaction, adherence, and, crucially, ameliorating diabetes management.

## Data Availability

The raw data supporting the conclusions of this article will be made available by the authors, without undue reservation.
