# Supplementary material for: Exploring usability metrics in continuous glucose monitoring systems: insights from the voice of people with diabetes in Italy
Source: Front Clin Diabetes Healthc. 2025 Mar 13;6:1472471. doi: 10.3389/fcdhc.2025.1472471 (PMC11966498; doi:10.3389/fcdhc.2025.1472471)
Supplement: Supplementary file 1 [file DataSheet1.docx]

Supplementary Material

Questionnaire submitted to Italian diabetic patients

**Part 1 – personal and medical background**

1. Regarding diabetes, you are:

- Person with diabetes
- Caregiver of a person with diabetes

1. Which gender do you identify with?

- Female
- Male
- None of the above
- Prefer not to say

1. Please indicate your age group:

- Under 18
- 18-24
- 25-34
- 35-44
- 45-54
- 55-64
- Over 65

1. What is your highest level of education?

- Postgraduate/Doctorate
- Master’s Degree (5 years)
- Bachelor’s Degree (3 years)
- High School Diploma
- Primary/Secondary School Certificate

1. What is your primary occupation?

- Executive/Manager
- Middle Manager
- Employee
- Worker/Laborer
- Merchant/Artisan
- Freelancer/Sales Representative
- Healthcare Professional
- Teacher
- Sales Assistant
- Bartender/Waiter
- Driver
- Student
- Retired
- Homemaker
- Unemployed
- Other (*please specify*)

1. Indicate your region of residence: [List of 20 Italian regions]
2. What type of diabetes are you affected by?

- Type 1
- Type 2
- Gestational diabetes
- Other (*please specify*)

1. How long ago were you diagnosed with diabetes?

- Less than 1 year
- 1 to 5 years
- More than 5 years

1. Does the treatment of your diabetes involve insulin administration?

- Yes
- Basal insulin only
- No, the treatment involves oral or non-insulin injectable drugs
- No, the treatment involves a specific diet

1. What tool do you use for blood glucose monitoring?

- I use a glucometer
- I use a continuous glucose monitoring (CGM) sensor/device
- I use a CGM sensor/device but also a glucometer
- I do not use any glucose monitoring tool

*[If sensor or sensor + glucometer]*

1. Please indicate the type of sensor you use:

- I use a Real-Time CGM device
- I use a Flash Glucose Monitoring (FGM) device
- Other (*please specify*)

1. How long have you been using this sensor?

- Less than 6 months
- 6 months to 1 year
- 1 to 2 years
- More than 2 years

1. How confident are you in relying on a sensor and app for daily monitoring of your health?

- Not confident at all
- Slightly confident
- Moderately confident
- Very confident
- Extremely confident

**Part 2 – Sensor features (for CGM system users)**

**How important are the following CGM sensor features to you?**

*(1 – not important at all; 2 – slightly important; 3 – fairly important; 4 – very important; 5 – extremely important)*

1. Size (area occupied by the sensor)

- 1
- 2
- 3
- 4
- 5

1. Thickness

- 1
- 2
- 3
- 4
- 5

1. Weight

- 1
- 2
- 3
- 4
- 5

1. Ability to place the sensor in different body positions

- 1
- 2
- 3
- 4
- 5

1. Waterproofness

- 1
- 2
- 3
- 4
- 5

1. Ability to personalize the aesthetic appearance of the sensor (e.g., with stickers)

- 1
- 2
- 3
- 4
- 5

**Ease of use**

*(1 – extremely difficult; 2 – difficult; 3 – neither easy nor difficult; 4 – easy; 5 – extremely easy)*

1. How easy was it to apply the sensor?

- 1
- 2
- 3
- 4
- 5

1. Think about the first time you configured the sensor parameters. How would you rate this activity?

- 1
- 2
- 3
- 4
- 5

1. Think about the first time you connected the sensor to your smartphone. How would you rate this activity?

- 1
- 2
- 3
- 4
- 5

**Please evaluate how you feel about always wearing a sensor:**

*(1 – not at all; 2 – slightly; 3 – fairly; 4 – significantly; 5 – extremely)*

1. Cared for

- 1
- 2
- 3
- 4
- 5

1. Safe

- 1
- 2
- 3
- 4
- 5

1. Protected

- 1
- 2
- 3
- 4
- 5

1. Anxious

- 1
- 2
- 3
- 4
- 5

1. Troubled

- 1
- 2
- 3
- 4
- 5

1. Judged

- 1
- 2
- 3
- 4
- 5

1. Thinking about your overall experience with the sensor, indicate your level of satisfaction:

*(1 – not satisfied at all; 2 – slightly satisfied; 3 – fairly satisfied; 4 – very satisfied; 5 – extremely satisfied)*

- 1
- 2
- 3
- 4
- 5

**Part 3 – System Usability Scale (for CGM system users)**

*(1 – not at all; 2 – slightly; 3 – fairly; 4 – significantly; 5 – extremely)*

1. Do you think you would like to use the application more frequently?

- 1
- 2
- 3
- 4
- 5

1. Do you find the application unnecessarily complex?

- 1
- 2
- 3
- 4
- 5

1. Do you find the application easy to use?

- 1
- 2
- 3
- 4
- 5

1. Do you think having expert technical support would be helpful for using the application more effectively?

- 1
- 2
- 3
- 4
- 5

1. Do you find that the various features of the application are well integrated?

- 1
- 2
- 3
- 4
- 5

1. Do you find significant inconsistencies between the application’s features?

- 1
- 2
- 3
- 4
- 5

1. Do you think most people can learn to use the application very quickly?

- 1
- 2
- 3
- 4
- 5

1. Do you find the application cumbersome/inconvenient to use?

- 1
- 2
- 3
- 4
- 5

1. Did you feel very confident while using the application?

- 1
- 2
- 3
- 4
- 5

1. Did you need to try using the application multiple times before mastering it?

- 1
- 2
- 3
- 4
- 5

**Part 4 – App functions (for CGM system users)**

**Indicate your habitual frequency of using the following features (if available in the sensor app you use):**

*(1 – never; 2 – rarely; 3 – sometimes; 4 – often; 5 – always)*

1. Compatibility with iOS (Apple) devices

- 1
- 2
- 3
- 4
- 5

1. Compatibility with Android devices

- 1
- 2
- 3
- 4
- 5

1. Display of actual blood glucose value

- 1
- 2
- 3
- 4
- 5

1. Display of glucose trends (e.g., trend arrows)

- 1
- 2
- 3
- 4
- 5

1. Adjustable thresholds for hypo/hyperglycemia

- 1
- 2
- 3
- 4
- 5

1. Countdown for warm-up session

- 1
- 2
- 3
- 4
- 5

1. Information/guide on sensor and transmitter (e.g., expiration date, replacement instructions)

- 1
- 2
- 3
- 4
- 5

1. Display of additional significant data (e.g., historical glucose values, GMI, time in range, etc.)

- 1
- 2
- 3
- 4
- 5

1. Ability to share monitoring data with your doctor

- 1
- 2
- 3
- 4
- 5

1. Ability to set alarms for monitored data

- 1
- 2
- 3
- 4
- 5

1. Ability to set predictive alarms

- 1
- 2
- 3
- 4
- 5

1. Alarm mode for signal loss

- 1
- 2
- 3
- 4
- 5

1. “Snooze”/Alarm delay feature

- 1
- 2
- 3
- 4
- 5

1. Voice commands (e.g., for visually impaired users)

- 1
- 2
- 3
- 4
- 5

1. Compatibility with smartwatches

- 1
- 2
- 3
- 4
- 5

1. Note-taking feature

- 1
- 2
- 3
- 4
- 5

1. Follower mode

- 1
- 2
- 3
- 4
- 5

**Part 5 – Building of the ideal CGM application and app satisfaction (for CGM system users)**

1. Imagine you could design a new app and had to choose only 3 features to keep. Which ones would you choose? [*Select 3 features*]

- Compatibility with iOS (Apple) devices
- Compatibility with Android devices
- Display of actual blood glucose value
- Display of glucose trends (e.g., trend arrows)
- Adjustable thresholds for hypo/hyperglycemia
- Countdown for warm-up session
- Information/guide on sensor and transmitter (e.g., expiration date, replacement instructions)
- Display of additional significant data (e.g., historical glucose values, GMI, time in range, etc.)
- Ability to share monitoring data with your doctor
- Ability to set alarms for monitored data
- Ability to set predictive alarms
- Alarm mode for signal loss
- “Snooze”/Alarm delay feature
- Voice commands (e.g., for visually impaired users)
- Compatibility with smartwatches
- Note-taking feature
- Follower mode

**How important is it to you?**

*(1 – not important at all; 2 – slightly important; 3 – fairly important; 4 – very important; 5 – extremely important)*

1. Receiving advice on the insulin dose to administer directly within the app?

- 1
- 2
- 3
- 4
- 5

1. Viewing the amount of active insulin in the app?

- 1
- 2
- 3
- 4
- 5

1. Having a function in the app to track daily activities?

- 1
- 2
- 3
- 4
- 5

1. Which relevant activities should be tracked in the app?

- Physical activity: typology (e.g., aerobic/anaerobic) and duration
- Nutrition: type of meal (e.g., portion/weight, carbohydrates, etc.)
- Hydration
- Administered insulin: type and dose
- Other (*please specify*)

1. Thinking about your overall experience with the app, indicate your level of satisfaction:

*(1 – not satisfied at all; 2 – slightly satisfied; 3 – fairly satisfied; 4 – very satisfied; 5 – extremely satisfied)*

- 1
- 2
- 3
- 4
- 5

1. Thinking about your overall experience with the sensor and the app, indicate the statement that best describes the impact on your quality of life:

*(1 – extremely negative impact; 2 – negative impact; 3 – no impact; 4 – positive impact; 5 – extremely positive impact)*

- 1
- 2
- 3
- 4
- 5
